# Health Professionals’ Perspectives on the Provision of Mental Health Services for People with Mental Health Problems and HIV: A Qualitative Study

**DOI:** 10.3390/healthcare14152351

**Published:** 2026-08-02

**Authors:** Malerotholi Thabida Posholi Mokokolisi, Winnie Baphumelele Ngcobo

**Affiliations:** School of Nursing & Public Health, The University of KwaZulu-Natal, Durban 4000, South Africa; ngcobow@ukzn.ac.za

**Keywords:** HIV, mental health services, mental disorders, primary health care, health personnel, qualitative research

## Abstract

**Background:** Mental health problems are common among people living with HIV (PLWH); however, the use of mental health services remains inadequate in many low- and middle-income countries. **Objectives:** This study aimed to explore the perspectives of health professionals on the provision of mental health services for PLWH presenting with mental health problems in primary health care in Lesotho. **Methods:** A qualitative descriptive approach was adopted, involving semi-structured interviews with 50 health professionals who were purposely selected, including nurses and medical officers, each with a minimum of 2 years’ primary health care experience in the Maseru district. Data were collected between December 2022 and January 2023, audio-recorded, and transcribed verbatim. Data collection continued until thematic saturation was achieved, and analysis was conducted using a thematic framework analysis. **Results:** The analysis revealed two main themes: (1) limited knowledge of mental health problems, encompassing an inadequate understanding of signs and symptoms, diagnosis, and management, and (2) barriers to the management of mental health problems. Key subthemes included the shortage of qualified mental health specialists and psychiatric medications, the absence of dedicated counselling services for the prevention of mental health problems, lack of a mental health department, time constraints within clinical practice, and limitations in clinical infrastructure. Cultural beliefs, stigma, and discrimination, both among health professionals and in the broader community, were also identified as significant impediments. Participants described a substantial treatment gap, driven by insufficient training and limited resources, which undermines the effective delivery of mental health services. **Conclusions:** The findings revealed that health professionals working in primary health care in Lesotho experience considerable knowledge gaps and systemic constraints that hinder the provision of adequate mental health services for PLWH who have mental health problems. These challenges can be mitigated through targeted capacity building, the integration of MHS into primary care, improved resource allocation, and strategies to reduce stigma, thereby enhancing health outcomes.

## 1. Introduction

Mental health problems (MHPs) are a significant global burden, especially for people living with HIV (PLWH), where comorbidity increases the risk of mortality, worsens HIV progression, and decreases adherence to antiretroviral therapy (ART) [1,2]. Depression prevalence among PLWH in sub-Saharan Africa (SSA), which bears more than two-thirds of the world’s HIV burden, ranges from 13% to 24%, with higher rates seen in women and young people [3,4,5]. In comparison to HIV-negative populations, PLWH have much higher rates of common mental disorders (CMDs), such as anxiety and depression, which can lead to worse HIV care continuum outcomes like delayed linkage to care and viral non-suppression [6,7]. Additionally, substance abuse and post-traumatic stress disorders co-occur with these disorders, making treatment compliance and quality of life even more challenging [8]. Depression is one of the most prevalent MHPs in PLWH but is undertreated in SSA [9]. Depressive symptoms in PLWH on ARVs range from 13% to 78% globally [10].

Treatment inconsistency for MHPs remains profound in low- and middle-income countries (LMICs), where over 75% of individuals with mental disorders receive no care, driven by limited resources, stigma, and inadequate integration into primary health care (PHC) [11]. For PLWH, untreated MHPs hinder achievement of the UNAIDS 95-95-95 targets, as mental distress impairs engagement across the HIV cascade [12]. Integration of mental health services (MHS) into HIV care has been advocated as a strategy to address this gap, leveraging existing HIV infrastructure to deliver screening, counselling, and pharmacotherapy through task-sharing with non-specialist providers [13,14]. The World Health Organisation’s Mental Health Gap Action Programme (mhGAP) Intervention Guide provides evidence-based protocols for non-specialists to manage priority disorders in PHC facilities, with updated reviews confirming its feasibility and impact in LMICs when supported by training and supervision [7,15]. The aim of the study was therefore to explore the perspectives of health professionals on the provision of MHS for PLWH presenting with MHPs in PHC facilities in Lesotho.

In SSA, barriers to MHS integration into HIV programs include health worker knowledge deficits, stigma among providers and communities, medication shortages, infrastructure limitations, and workloads, and community sensitisation [13,16,17]. Recent studies have highlighted persistent challenges in task-sharing models, with provider attitudes and resource constraints hindering scale-up [18,19]. In Lesotho, a high-HIV-burden country, nurses report uncertainty about the HIV–mental health nexus and lack competency for integration, underscoring systemic limitations [20]. Patient preferences in Lesotho f avour face-to-face, confidential, health worker-led behavioural services, yet access remains constrained by stigma and service gaps [1]. PHC facilities in Lesotho deliver the majority of HIV services, yet MHS provision for PLWH presenting with MHPs is minimal. Health professionals frequently encounter patients with comorbid conditions but report inadequate knowledge for identification and management, compounded by absent specialised staff, limited psychiatric medications, and infrastructural barriers. This may contribute to unmet needs, suboptimal ART adherence, and increased risk of adverse HIV-related outcomes. Although regional studies document high MHP comorbidity in PLWH and general barriers to MHS integration in SSA [3,8], context-specific insights from health professionals in Lesotho are scarce. To our knowledge, limited qualitative evidence has explored provider perspectives on MHS delivery for PLWH with MHPs at the PHC level in this setting, limiting targeted interventions to address local implementation challenges.

Exploring health professionals’ perspectives is essential to inform strategies for integrating MHS into HIV care in Lesotho, aligning with mhGAP recommendations and national HIV priorities [15]. Such evidence can guide training, policy advocacy, and resource allocation to reduce the treatment gap, enhance ART outcomes, and support sustainable scale-up of integrated services. This study explored health professionals’ perspectives on the provision of MHS for PLWH presenting with MHPs.

## 2. Methods

### 2.1. Study Design, Setting, and Participants

This study employed a qualitative descriptive design to explore health professionals’ perspectives on the provision of MHS for people with MHPs and HIV in PHC facilities. A qualitative descriptive approach was selected, as it is particularly suited for providing a straightforward description of phenomena from the participants’ viewpoints, without the imposition of preconceived theoretical frameworks, allowing for an in-depth understanding of real-world experiences and barriers in healthcare delivery [21]. This design aligns with the study’s aim to capture nuanced perceptions that quantitative methods might overlook, thereby facilitating the identification of themes directly relevant to policy and practice improvements in resource-limited settings, such as Lesotho.

The study was grounded in a constructivist/phenomenological orientation, which posits that reality is constructed through individuals’ lived experiences and interactions [22,23]. This orientation justified the use of semi-structured interviews, as it enabled participants to express their perspectives in their own words, promoting a deeper exploration of subjective realities.

The study was conducted in the Maseru district, Lesotho, specifically within five PHC facilities. This setting was chosen because Maseru represents a mix of urban and peri-urban environments where HIV prevalence is high, and PHC serves as the primary entry point for healthcare, including potential MHS integration. To our knowledge, limited studies on MHS for PLWH in Lesotho were identified, underscoring the need for this investigation in this context.

Participants included registered nurses and medical doctors with at least two years of experience in PHC facilities, and if hired on a contract, being hired for two years was maintained. These health professionals were chosen because they were believed to have more experience in HIV and mental health (MH) care. The participants who were not willing to be audio-recorded were excluded from the interview A purposive sampling strategy was utilised to deliberately select “information-rich” individuals who could provide detailed insights into the research topic [24,25]. This non-probability approach was justified over random sampling, as it prioritises depth over generalizability, targeting those directly involved in PHC service delivery. After receiving approval to conduct the study, letters were sent to all PHC facilities to request permission to conduct the study. A list of eligible health professionals was obtained from each PHC facility manager, and participants were invited to an informational meeting where the study was explained, and the date and time for interviews were obtained. The participants finally signed the written informed consent before data collection. Recruitment continued until no new codes could be obtained, resulting in a sample of 50 participants (38 nurses and 12 medical doctors), which is considered adequate for thematic saturation in qualitative studies of this nature [26].

### 2.2. Study Instruments

Data were collected using a semi-structured interview schedule developed by researchers, consisting of 14 open-ended questions guided by the study’s aim to explore perspectives on MHS provision. The schedule included prompts on knowledge of MHPs, barriers to service delivery, and management strategies, allowing flexibility for follow-up questions based on participants’ responses. This format was chosen to strike a balance between structure and openness, encouraging rich, narrative data while ensuring comprehensive coverage of key topics [27]. The interview schedule was informed by a review of relevant literature on MHPs in PLWH and barriers to MHS integration. To enhance quality and validity, the instrument was piloted with five nursing managers from similar PHC facilities, as they were believed to have experience in HIV and MH care prior to being nursing managers in the same facilities (not included in the final sample). The research instrument only required minor refinements for clarity and flow after the pilot study. It was reviewed by two experts, one on MH and one in qualitative research methods, to ensure content relevance and methodological rigour.

Interviews were conducted in English by the researcher, as all the participants had reached tertiary education; therefore, the researcher believed that the participants were familiar with English. The interviews were conducted in private rooms at the PHC facilities, lasting 45–60 min each, and audio-recorded with participants’ permission to capture verbatim responses accurately. Field notes were also taken to document non-verbal cues and contextual observations. Data collection took place from December 2022 to January 2023, a period chosen to minimise peak holiday disruptions while aligning with PHC availability.

### 2.3. Confidentiality and Ethical Considerations

Ethical approval was obtained from the Biomedical Research Ethics Committee (BREC) at the University of KwaZulu-Natal (protocol number: BREC/00004710/2022), as well as from the Ministry of Health in Lesotho and the management of the participating PHC facilities. All procedures adhered to the ethical principles outlined in the Declaration of Helsinki, emphasising respect for persons, beneficence, and justice.

Written informed consent was secured from each participant after providing a detailed information sheet explaining the study’s purpose, procedures, potential risks (e.g., emotional discomfort discussing sensitive topics), benefits (e.g., contributing to improved MHS), and their rights, including voluntary participation and the ability to withdraw at any time without repercussions. Participants were assured of confidentiality; no personal identifiers were used in the data collection or reporting process. Audio recordings and transcripts were stored securely on password-protected devices, accessible only to the research team, with plans to delete them after five years, as per institutional guidelines. Anonymity was maintained through numerical coding of responses.

To mitigate potential power imbalances, the lead researcher conducted the interviews, maintaining a non-supervisory role over the participants. Debriefing was offered after the interview, and referrals to counselling services were available if distress arose. The study prioritised cultural sensitivity, given the stigma around MHPs and HIV in Lesotho, by framing questions neutrally and respecting local norms.

### 2.4. Data Analysis

Thematic framework analysis was employed to analyse the qualitative data, following the steps outlined by [28]: familiarisation, coding, theme development, reviewing, defining, and reporting. This method was selected for its systematic yet flexible approach, which is suitable for applied qualitative research aiming to inform policy, as it allows for both deductive (guided by the research aim) and inductive (emerging from the data) theme identification.

Interviews were transcribed verbatim by the lead researcher, and the transcripts were reviewed for accuracy against the audio recordings by an independent research expert to enhance the trustworthiness of the findings. Initial coding was conducted independently by two coders (the lead researcher and a qualitative expert) to identify patterns, ensuring inter-coder reliability through consensus discussions on discrepancies. Codes were grouped into subthemes and overarching themes, with constant reference to the study’s objectives [27].

Trustworthiness was upheld using [29] criteria: credibility (through prolonged engagement, member checking via summary feedback to a subset of participants, and peer debriefing); transferability (via thick descriptions of context); dependability (through an audit trail of field notes, recordings, and decisions); and confirmability (by reflexivity journaling to minimise researcher bias). Data saturation was confirmed when no new themes emerged from additional interviews.

## 3. Results

The findings revealed that the sample comprised predominantly females (n = 36), with males accounting for a smaller proportion (n = 14). Participants’ ages ranged from 24 to 52 years, with a mean age of 34.34 (M = 34.34. SD = 6.74, range = 24–52), indicating a broad representation of health professionals across the early- to late-career spectrum. All participants had at least 2 years of experience working in PHC, with length of service ranging from 2 to 18 years. Most participants were Registered Nurse Midwives, reflecting their central role in PHC service delivery. Other cadres included medical officers, registered ophthalmic nurses, registered nurse clinicians, and nursing officers (see Table 1 for details).

### 3.1. Themes

Two themes emerged from the analysis: (1) knowledge of MHPs, and (2) management of MHPs in people presenting with MHPs and HIV.

### 3.2. Knowledge of MHPs (Theme 1)

Three subthemes emerged related to the knowledge of MHPs:

- Limited knowledge regarding the signs and symptoms of MHPs;
- Limited knowledge regarding the diagnosis of MHPs;
- Limited knowledge regarding the management of MHPs.

The qualitative findings indicated that the majority of health professionals reported being unfamiliar with the management of MHPs. In addition, most participants reported having limited knowledge regarding the signs, symptoms, and diagnosis of MHPs. As a result, most participants indicated that they had difficulty in identifying other MHPs except psychosis, which can be identified even by lay personnel in health.

*I did not know that MHPs and HIV are closely related. These services are not offered mainly due to a lack of knowledge concerning MH*. (P16)

*We last did MH at the college, so we are out of information concerning psychiatric, so we should be trained on MH so that these services can be provided*. (P43)

*You know, I think we meet so many psychiatric patients that we fail to address because sometimes I suspect that this might be anxiety. However, because I have forgotten almost everything about psychiatric, I fail to make informed decision then leave the patient without assisting the patient regarding her problem because I did not have information to say this might be a specific condition in psychiatric*. (P34)

### 3.3. Management of MHPs in People Presenting with MHPs and HIV (Theme 2)

Six subthemes related to the management of MHPs in people presenting with MHPs and HIV:

- No availability of psychiatric medication;
- No mental health professional to consult at the facility;
- No employed MH professional, such as a psychologist, psychiatric nurse or psychiatrist;
- No MH department in PHC; consequently, MHS are not offered;
- No counselling services are offered to patients with HIV to prevent MHPs;
- No adequate time to manage mental health problems, clinic structure, stigma and discrimination, and cultural factors seems to be a problem.

The qualitative findings indicated that the majority of health professionals reported the unavailability of qualified health professionals in MH at PHC facilities and a need for at least one MH professional to visit PHCs weekly. Most participants were of the view that at least one psychiatric nurse should be hired in PHC facilities in order to respond to the growing MHPs in PHC facilities.

*There are no people skilled in MH at the PHC facilities, so it is not easy for MHS to be offered at the PHC*. (P39)

*Shortage of specialists on MH is a central problem, as we are not specialists on MH and we cannot fully know about mental health*. (P43)

*There are no specialists on MH in PHC, yet it is a crucial factor for MHS to be offered at the PHC*. (P16)

*The unavailability of specialists on MH in PHC causes a significant treatment gap*. (P21)

*There is a need for MH professionals to do weekly visits in PHC*. (P24)

Some participants also indicated the issue of the unavailability of psychiatric medication and a psychiatric department in the PHC facilities, where such services could be offered. Most participants indicated they did not even remember psychiatric medication, as they last saw the drugs during their college time. Having a psychiatric department in PHC facilities was viewed as a great need.

*There are no psychiatric department and there are very few psychiatric medications in PHC facilities to the extent that even the continuity of care is complicated for people who were admitted at Mohlomi and therefore need to continue with care in the nearest PHC*. (P41)

*I only saw a few psychiatric drugs here, and they are rarely prescribed to patients*. (P26)

Most participants were of the view that the counselling services that are offered to clients with HIV are mainly adherence counselling about antiretroviral drugs, not counselling to assist patients to accept their status to prevent MHPs. Most participants were of the view that social workers are not skilled enough when it comes to counselling skills, as their work entails more supporting clients socially.

*There are counselling services offered by social workers in the facility, but what I realised is that the counselling is mainly on drug adherence*. (P28)

*The counselling services in this facility are offered by social workers, so they are not skilled enough to provide thorough counselling, given their level of knowledge*. (P15)

*I believe counselling services regarding prevention of MHPs are very minimal in this facility, as most counselling that is done by social workers is mainly on adherence to ARVs*. (P24)

Some health professionals also indicated the issue of time constraints, which affects the delivery of MHS. Most participants who were medical doctors were of the view that psychiatric assessment requires a lot of time, which they do not have in PHC facilities. Therefore, time constraints were seen as a great barrier to MHS in PHC facilities.

*Psychiatric assessment needs a longer time, so I cannot manage to see one patient for an hour while I am supposed to see around 40 patients or more in a day*. (P17)

*When you see an MH patient, other patients become impatient and start to knock at the door*. (P37)

*If I take a longer time with one patient in here, you will see other patients knocking at the door to show you that I have delayed*. (P3)

A few nurses said the way the clinic infrastructure was currently set up did not allow for comprehensive services.

*You know, even if we like to provide these services, we don’t have space where we can say this is a place where people with MHPs can be helped because the space is just too small*. (P21)

*There is not enough space to see MHP’s client at the PHC*. (P18)

A few participants also indicated that there must be severe stigma among health professionals who are stake holders concerning MHPs, as they do nothing with regard to MH needs in Basotho. In addition to this, most participants indicated that stake holders only focus on conditions such as HIV, Aids, and tuberculosis because they have funding.

*What is surprising is that the Director for Nursing in Lesotho is a psychiatric nurse, but MHS are not improving because MH is neglected*. (P36)

*MHPs are stigmatised by everyone, policymakers and the community at large, so it’s not easy for these services to be fully delivered therefore it’s not so easy to have resources to fund MH as a stigmatised health problem*. (P23)

*Until stakeholders understand MH, it will not be easy for MHS to be offered*. (P24)

## 4. Discussion

This qualitative descriptive study explored health professionals’ perspectives on the provision of MHS for PLWH who present with MHPs in PHC facilities in Maseru, Lesotho. The findings reveal two primary themes: limited knowledge of MHPs, and multifaceted barriers to their management. These insights highlight a critical treatment gap in a high-HIV-burden setting, where approximately 80% of PLWH experience comorbid MHPs, yet access to integrated care remains severely restricted [3,4]. The first theme, limited knowledge of MHPs, including inadequate recognition of signs and symptoms, diagnostic challenges, and management strategies, emerged as a foundational barrier. Participants frequently described outdated training from nursing or medical education, leading to hesitation in addressing suspected MHPs during HIV consultations. This aligns with regional evidence from sub-Saharan Africa (SSA), where up to 90% of PLWH with depression go undiagnosed due to primary care providers’ insufficient mental health literacy [2,8]. A recent scoping review of interventions integrating MHS into HIV care across Africa identified under diagnosis as a core issue, exacerbated by the absence of routine screening tools in PHC [26]. In Lesotho, nurses’ uncertainty about the HIV–MHP nexus, as reported here and in prior local surveys [17], perpetuates inaction, mirroring knowledge gaps observed in similar resource-limited contexts like Nepal, where primary care workers cited comparable training deficiencies [13]. These deficits not only delay intervention but also contribute to multiple harms: untreated MHPs impair antiretroviral therapy (ART) adherence, while HIV-related stigma amplifies psychological distress [12].

The second theme encompassed systemic and sociocultural barriers to MHP management, including shortages of qualified MH specialists and psychiatric medications, absence of dedicated counselling for MHP prevention in PLWH, lack of MH departments, time constraints from high patient loads, infrastructural limitations, and pervasive stigma/discrimination. Participants’ calls for weekly specialist outreach reflect a desire for task-sharing models, yet current PHC structures prioritise HIV adherence counselling over holistic MH support. These challenges are emblematic of SSA-wide impediments, where human resource shortages, one psychiatrist per 500,000 people in many countries, coupled with medication stockouts, undermine integration efforts [5,7]. A multimethod study in the Democratic Republic of the Congo similarly documented low prioritisation of MH, irregular psychotropic supplies, and inadequate infrastructure as key health system barriers, with stigma deterring both provider engagement and patient uptake [30]. In Lesotho, urban PHC facilities like those studied here face additional pressures from PHC overcrowding, echoing findings from ART adherence reviews that link workload burdens to fragmented care [31]. Stigma, manifesting as provider neglect or community discrimination, further entrenches inequities, consistent with cross-cultural analyses that show health workers’ internalised biases as a deterrent to MHS delivery [1,6,11]. Local initiatives, such as Partners in Health’s integration of depression screening into HIV/TB services, demonstrate feasibility but reveal gaps in scale-up, particularly for preventive counselling [18].

These barriers sustain a vicious cycle wherein untreated MHPs, prevalent in 13–24% of PLWH in SSA, with higher rates among women and youth, exacerbate ART non-adherence, viral non-suppression, and HIV transmission risks [2,4,23]. A meta-analysis of 90 studies confirmed that integrated HIV–PHC models improve outcomes, such as retention and viral load suppression; however, implementation is hindered by the very resource constraints identified here [32].

This study’s strengths lie in its novelty as the first in-depth qualitative exploration of provider perspectives on MHS for PLWH with MHPs in Lesotho, filling a critical evidence void in a nation with the world’s second-highest HIV prevalence. The purposive sample of 50 participants from five clinics achieved thematic saturation, supported by rigorous thematic framework analysis, inter-coder reliability, and adherence to trustworthiness criteria.

## 5. Limitations

Limitations include the focus on urban/peri-urban Maseru PHC facilities, which may limit transferability to rural Lesotho, where geographic barriers are more pronounced. Data from late 2022 to early 2023 may not accurately capture post-pandemic shifts, and self-reported perspectives could introduce social desirability bias; however, anonymity and neutral probing helped mitigate this.

## 6. Conclusions

The findings revealed that substantial knowledge gaps, unavailability of psychiatric medication, unavailability of MH professionals, unavailability of a psychiatric department in PHC facilities, stigma and discrimination from health professionals and stake holders regarding MHPs and systemic barriers continue to impede the delivery of MHS for PLWH with MHPs in Lesotho’s PHC facilities, perpetuating a significant treatment gap and limiting progress toward the UNAIDS 95–95–95 targets. Urgent integration of MH into HIV services is required, supported by mhGAP-aligned refresher training, task-shifting with specialist outreach, strengthened medication supply and infrastructure, and targeted stigma-reduction initiatives for health professionals. Future research should incorporate the perspectives of people living with HIV, evaluate the effectiveness of integrated care models such as Partners in Health, supported approaches, and assess the impact of mental health integration on key HIV outcomes. Prioritising MH within national HIV strategies remains essential for improving care quality and advancing equitable epidemic control in Lesotho and across sub-Saharan Africa.

## Figures and Tables

**Table 1:** The demographic information of the sample.

Interview ID	Gender	Period of Work in PHC	Age (Years)	Occupational Position
01	F	3 years	29	Registered nurse midwife
02	F	12 years	35	Registered nurse midwife
03	M	6 years	36	Medical officer
04	F	5 years	29	Registered nurse midwife
05	M	2 years	26	Registered nurse midwife
06	F	12 years	45	Medical officer
07	F	2 years 9 months	28	Registered nurse midwife
08	F	3 years	34	Registered nurse midwife
09	M	2 years	27	Registered nurse midwife
10	F	4 years	28	Registered nurse midwife
11	F	11 years	38	Registered nurse midwife
12	F	11 years	39	Registered nurse midwife
13	F	2 years 9 months	24	Registered nurse midwife
14	F	9 years	33	Registered nurse midwife
15	F	8 years	34	Registered nurse midwife
16	M	7 years	43	Registered nurse midwife
17	M	2 years	29	Medical officer
18	M	9 years	45	Medical officer
19	F	10 years	38	Registered ophthalmic nurse
20	F	18 years	46	Registered ophthalmic nurse
21	F	2 years	29	Registered ophthalmic nurse
22	M	2 years 1 month	25	Registered nurse midwife
23	F	6 years	39	Registered ophthalmic nurse
24	F	7 years	41	Nursing officer
25	F	11 years	35	Registered nurse midwife
26	F	2 years	24	Registered nurse midwife
27	M	2 years	30	Registered nurse midwife
28	F	6 years	31	Registered nurse clinician
29	F	2 years 8 months	29	Registered nurse midwife
30	M	2 years	26	Registered nurse midwife
31	F	2 years 9 months	26	Registered nurse midwife
32	F	9 years	33	Registered nurse midwife
33	M	4 years	36	Registered nurse midwife
34	F	12 years	52	Registered nurse midwife
35	F	5 years	41	Registered nurse midwife
36	F	7 years	45	Registered nurse midwife
37	M	5 years	33	Medical officer
38	F	9 years	36	Registered nurse midwife
39	F	7 years	33	Registered nurse midwife
40	F	14 years	40	Registered nurse midwife
41	F	6 years	37	Registered nurse midwife
42	F	10 years	38	Registered nurse midwife
43	F	2 years	27	Registered nurse midwife
44	F	5 years	40	Registered nurse midwife
45	F	3 years	31	Medical officer
46	F	16 years	46	Nursing officer
47	F	8 years	35	Registered nurse midwife
48	M	4 years	36	Medical officer
49	M	7 years	31	Registered nurse
50	M	3 years	26	Registered nurse

M = Male, F = Female, PHC = Primary health care.

## Data Availability

All data are available to the researcher on reasonable request.

## Abbreviations

PLWH	People living with HIV
MHPs	Mental health problems
MH	Mental health
MHS	Mental health services
PHC	Primary health care
LMIC	Lower middle-income countries
CMD	Common mental disorders
MhGAP	Mental health gap action program
ART	Antiretroviral therapy

## References

[1] Yoon G.H., Johnson N.E., Mokebe M., Mahlatsi P., Lerotholi M., Labhardt N.D. (2025). Face-to-face, confidential and health worker-led: Understanding the preferences for behavioural health services among people with HIV in Lesotho. Camb. Prism. Glob. Ment. Health.

[2] Adjorlolo S., Ameyaw E.K., Adu-Gyamfi S., Attah P.O., Avege K., Ayisi-Addo S. (2025). Mental health interventions for young people living with HIV/AIDS in sub-Saharan Africa: A systematic review. AIDS Res. Treat..

[3] Boakye D.S., Amu H., Sebsibe T.A., Agyemang-Duah W., Adu C., Senkyire E.K. (2024). Mental health burden among females living with HIV and AIDS in sub-Saharan Africa: A systematic review. PLoS Glob. Public Health.

[4] Too E.K., Abubakar A., Nasambu C., Koot H.M., Cuijpers P., Newton C.R.J.C., Nyongesa M.K. (2021). Prevalence and factors associated with common mental disorders in young people living with HIV in sub-Saharan Africa: A systematic review. J. Int. AIDS Soc..

[5] Ahad A., Sánchez-González M., Junquera P. (2023). Understanding and addressing mental health stigma across cultures for improving psychiatric care: A narrative review. Cureus.

[6] Al-Hashemi T., Al-Mahrouqi T., Al-Huseini S., Al Salmi M., Al Nuumani R., Al Balushi F. (2025). Mental health stigma and help-seeking behaviours among primary healthcare physicians in Oman. Int. J. Environ. Res. Public Health.

[7] Keynejad R., Spagnolo J., Thornicroft G. (2021). WHO mental health gap action programme (mhGAP) intervention guide: Updated systematic review on evidence and impact. Evid.-Based Ment. Health.

[8] Komu C.K., Ngigi M., Melson A.J. (2025). Barriers and Facilitators to Accessing Mental Health Services for Adults in Sub-Saharan Africa: A Systematic Review. Ment. Health Sci..

[9] Bernard C., Dabis F., De Rekeneire N. (2017). Prevalence and factors associated with depression in people living with HIV in sub-Saharan Africa: A systematic review and meta-analysis. PLoS ONE.

[10] Beyene Gebrezgiabher B., Huluf Abraha T., Hailu E., Siyum H., Mebrahtu G., Gidey B., Abay M., Hintsa S., Angesom T. (2019). Depression among Adult HIV/AIDS Patients Attending ART Clinics at Town, Aksum, Ethiopia: A Cross-Sectional Study. Depress. Res. Treat..

[11] DeAngelis T. (2024). Fighting stigma by mental health providers toward patients. Monit. Psychol..

[12] Cele W., Mhlongo E. (2020). Health professionals’ perceptions of the integration of mental health into HIV services. Glob. J. Health Sci..

[13] Upadhaya N., Regmi U., Gurung D., Luitel N., Petersen I. (2020). Mental health and psychosocial support services in primary health care in Nepal: Perceived facilitating factors, barriers and strategies for improvement. BMC Psychiatry.

[14] Conteh N., Mahomed O. (2025). Patient and healthcare worker perspectives on the integration of mental health services into routine ART services in Windhoek, Namibia: A qualitative study. Int. J. Integr. Care.

[15] Conteh N., Latona A., Mahomed O. (2023). Mapping the effectiveness of integrating mental health in HIV programs: A scoping review. BMC Health Serv. Res..

[16] Febana B., Mulondo M. (2025). Perceptions of psychiatric-trained nurses on integrating mental health into primary health care in Africa. Health SA Gesondheid.

[17] Ramokanate S., Nyangu I., Rathobei L. (2023). Exploring nurses’ perceptions of integration of mental health services in HIV/AIDS treatment in Lesotho: A qualitative survey. New Voices Psychol..

[18] Partners In Health (2018). Treating Mind and Body: Mental Health Care Expanding in Lesotho. https://www.pih.org/article/treating-mind-and-body-mental-health-care-expanding-lesotho.

[19] Kwaitana D., Chisoni F., van Breevoort D., Mildestvedt T., Meland E. (2023). Primary healthcare service delivery for older people with progressive multimorbidity in low- and middle-income countries: A systematic review. Trans. R. Soc. Trop. Med. Hyg..

[20] Windle A., Javanparast S., Freeman T., Baum F. (2023). Factors that influence evidence-informed meso-level regional primary health care planning: A qualitative examination and conceptual framework. Health Res. Policy Systems.

[21] Sandelowski M. (2000). Whatever happened to qualitative description?. Res. Nurs. Health.

[22] Cohen L., Manion L., Morrison K. (2002). Research Methods in Education.

[23] Tashakkori A., Teddlie C. (2009). Handbook of Mixed Methods in Social & Behavioural Research.

[24] Grove S.K., Burns N.J.G. (2015). The Practice of Nursing Research: Appraisal, Synthesis, and Generation of Evidence.

[25] Botma Y., Greeff M., Mulaudzi F.M., Wright S.C. (2010). Research in Health Sciences.

[26] 25 Guest G., Bunce A., Johnson L. (2006). How many interviews are enough? An experiment with data saturation and variability. Field Methods.

[27] Kallio H., Pietilä A.M., Johnson M., Kangasniemi M. (2016). Systematic methodological review: Developing a framework for a qualitative semi-structured interview guide. J. Adv. Nurs..

[28] Gale N.K., Heath G., Cameron E., Rashid S., Redwood S. (2013). Using the framework method for the analysis of qualitative data in multi-disciplinary health research. BMC Med. Res. Methodol..

[29] Lincoln Y.S., Guba E.G. (1985). Naturalistic Inquiry.

[30] Mayoyo E., Chenge F., Sow A., Criel B., Michielsen J., Van den Broeck K. (2024). Health system facilitators and barriers to the integration of mental health services into primary care in the Democratic Republic of the Congo: A multimethod study. BMC Prim. Care.

[31] Magura J., Nhari S., Nzimakwe T. (2025). Barriers to ART adherence in sub-Saharan Africa: A scoping review toward achieving UNAIDS 95-95-95 targets. Front. Public Health.

[32] Goldstein D., Salvatore M., Ferris R., Phelps B., Minior T. (2023). Integrating global HIV services with primary health care. Lancet Glob. Health.

